# Precocious puberty secondary to a mixed germ cell-sex cord-stromal tumor associated with an ovarian yolk sac tumor: a case report

**DOI:** 10.1186/1752-1947-6-162

**Published:** 2012-06-26

**Authors:** Kotb Abbass Metwalley, Dalia Ahmed Elsers, Hekma Saad Farghaly, Hanaa Abdel-Lateif, Mohamed Abdel-Kader

**Affiliations:** 1 Paediatric Endocrinology Unit, Department of Paediatrics, Faculty of Medicine, Assiut University, Assiut, Egypt; 2 Department of Pathology, Faculty of Medicine, Assiut University, Assiut, Egypt; 3 Department of Pediatric Surgery, Faculty of Medicine, Assiut University, Assiut, Egypt

## Abstract

**Introduction:**

Ovarian tumors are the least common cause of sexual precocity in girls. Mixed germ cell-sex cord-stromal tumors associated with a yolk sac tumor of the ovary are rare neoplasms, of which only a small number of well-documented cases have been described so far. Here, we report precocious puberty in a four-year-old Egyptian girl caused by a mixed germ cell-sex cord-stromal tumor associated with a yolk sac tumor of the ovary.

**Case presentation:**

A four-year-old Egyptian girl was referred to our pediatric endocrinology unit for evaluation of bilateral breast budding, pubic hair and vaginal bleeding. On examination, we found that her breast enlargement and pubic hair were compatible with Tanner III. A thorough workup revealed a large mass in her right ovary. Magnetic resonance imaging ofher brain showed that her pituitary gland was normal. A hormonal assay revealed high levels of estradiol, 280 to 375pmol/L; progesterone, 5.3 nmol/L; testosterone 38.9 pg/mL; and androstenedione, 4.1 ng/mL. Her basal and stimulated levels of luteinizing hormone and follicle-stimulating hormone were low. Tumor markers levels were high, with a total inhibin of 1,069U/L and an alpha-fetoprotein of 987 μg/L. Her chromosomes were normal (46XX). Our patient underwent an explorative laparotomy and a solid tumor localized to her right ovary was identified. A right salpingo-oophorectomy was performed and the histopathological diagnosis was a mixed germ cell-sex cord-stromal tumorwith a yolk sac tumor of the ovary. Postoperatively, she was started on treatment with chemotherapy. Our patient is doing well without evidence of tumor recurrence or metastasis during eight months of postoperative follow-up.

**Conclusion:**

Although a mixed germ cell-sex cord-stromal tumor associated with a yolk sac tumor of the ovary is a rare occurrence, it should be considered in the differential diagnosis for a prepubescent girl with an abdominal mass and precocious puberty.

## Introduction

Precocious puberty in girls is defined by the development of sexual characters before the age of eight years. It is usually due to the premature activation of the hypothalamic-pituitary-ovarian axis, defined as central precocious puberty (CPP) [1]. It is rarely of ovarian or adrenal origin. CPP in girls is idiopathic in the majority of cases [2]. Ovarian tumors are the least common cause of sexual precocity. Functional neoplasms of the ovary are relatively infrequent and only 5% occur before puberty [3]. Granulosa-theca cell tumors are the most common ovarian tumors to cause precocious puberty in girls [4]. Most ovarian tumors develop from one of three sources: the germinal epithelium covering the urogenital ridge; the underlying stromal elements of the urogenital ridge; or germ cells from the yolk sac. The most common malignant germ cell tumors are dysgerminomas (48%) and yolk-sac tumors (20%). Approximately 10% of neoplasms are composed of a mixture of different histological types [5]. Tumors that contain an admixture of germ cell and sex cord-stromal derivation include gonadoblastoma and mixed germ cell-sex cord-stromal tumor (MGCCST) -non gonadoblastoma type [6]. To the best of our knowledge, an MGCCST with a yolk sac tumor has not yet been described in Egypt. Here, we describe the clinical, histopathological and immunohistological findings of a case of an MGCCST with a yolk sac tumor that occurred in a four-year-old Egyptian girl presenting with precocious puberty.

## Case presentation

A four-year-old Egyptian girl was referred to our pediatric endocrinology unit for evaluation of bilateral breast budding and growth of pubic hair of four months duration followed by two episodes of vaginal spotting, initially noticed two weeks prior to her presentation. Her previous medical history and birth history were unremarkable; there was no family history of endocrinological disorders or precocious puberty. At presentation, our patient was an alert, irritable child. Her height was 115 cm (>97th percentile) and weight 20 kg (90th percentile). Her vital signs were normal. She had normal ocular, thyroid and cardiorespiratory examinations and no lymphadenopathy. Her breast tissue was palpable and the contour of her nipples and areolae was equivalent to Tanner stage III breast development. Her pubic hair was coarse, pigmented and distributed over her labia majora, equivalent to Tanner stage III [7]. Inspection of her external genitalia revealed estrogenized vulval mucosa with a clear viscous discharge, and the clitoris was slightly enlarged. An abdominal mass was palpable in her suprapubic area, firm in consistency, not tender, with ill-defined borders. There was no obvious ascities.

**Figure 1:**
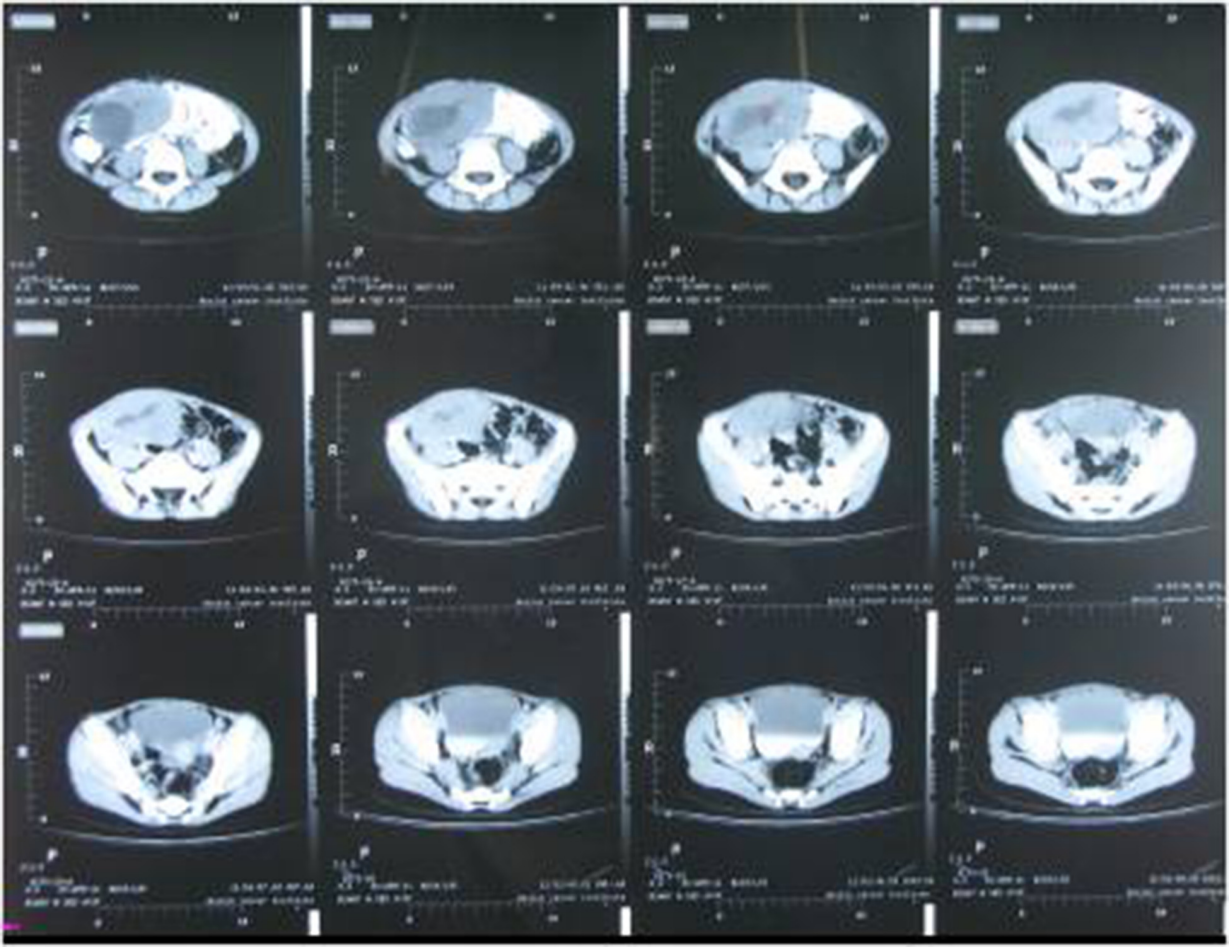
Computed tomography of the abdomen and pelvis shows a huge right ovarian mass.

An initial ultrasonographic study of her abdomen and pelvis revealed an enlarged uterus with prominent endometrium and 7.8 cm × 5.2 cm irregular, inhomogeneous mass in the right adnexal region lying above her uterus and crossing the midline. Her left ovary, adrenals, liver, spleen and kidneys were normal. Computed tomography imaging of her abdomen and pelvis confirmed the above mentioned findings (Figure 1). Subsequent testing of her brain by magnetic resonance imaging revealed a normal-sized pituitary and infundibulum with a normal posterior pituitary bright spot. An X-ray of her left hand and wrist revealed a bone age of 5 years according to female standards [8].

**Figure 2:**
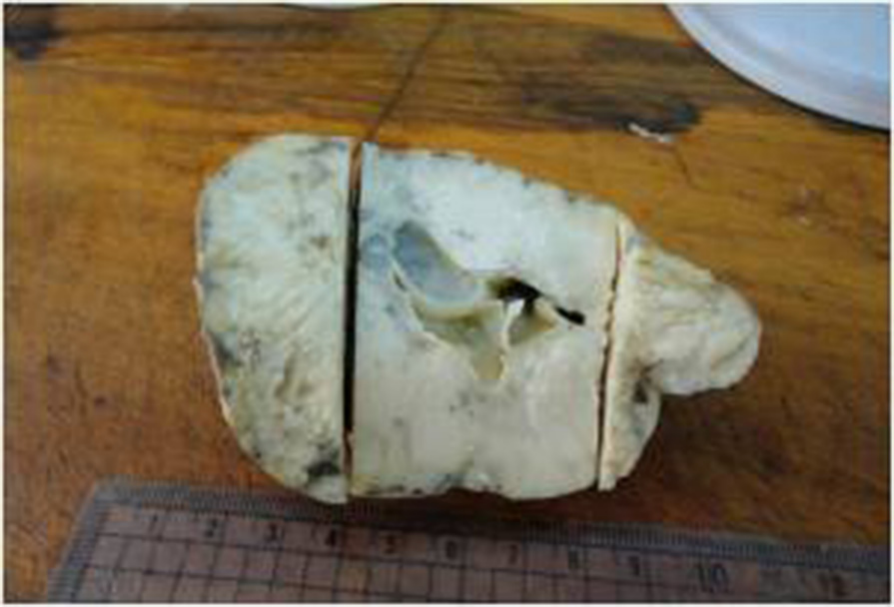
**The cut section was solid with multiple cysts containing transparent thick material.** The mass was firm in consistency with friable areas. Its color was yellow grayish with small dark areas of necrosis.

**Figure 3:**
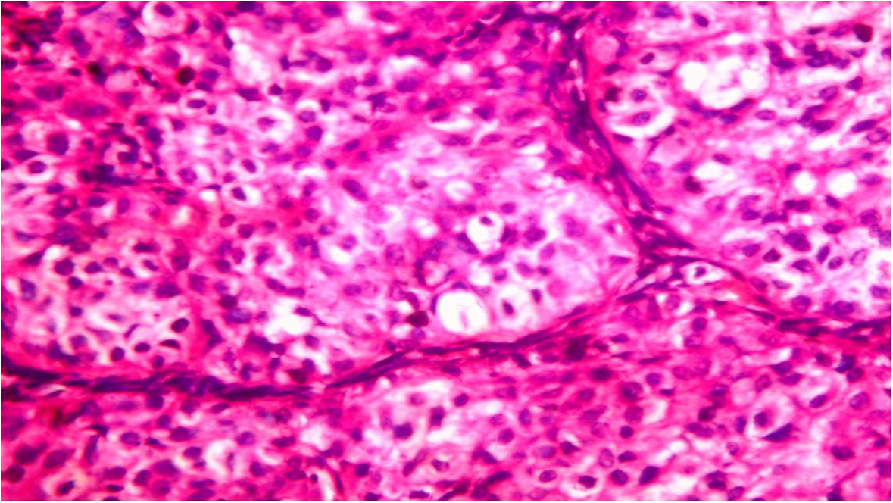
**Hematoxylin and eosin staining × 40.** Large polygonal cells with abundant eosinophilic cytoplasm, and rounded hyperchromatic nuclei growing in compact masses separated by thin fibrous bands.

**Figure 4:**
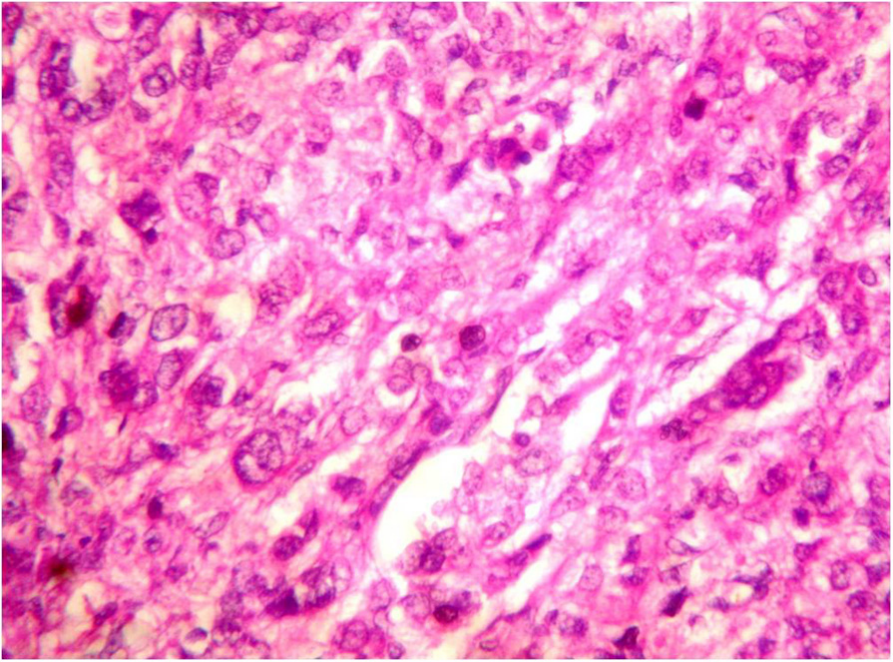
**Hematoxylin and eosin staining × 40.** Primitive tumor cells with vacuolated cytoplasm. Nuclei are rounded and hyperchromatic, with prominent nucleoli.

**Figure 5:**
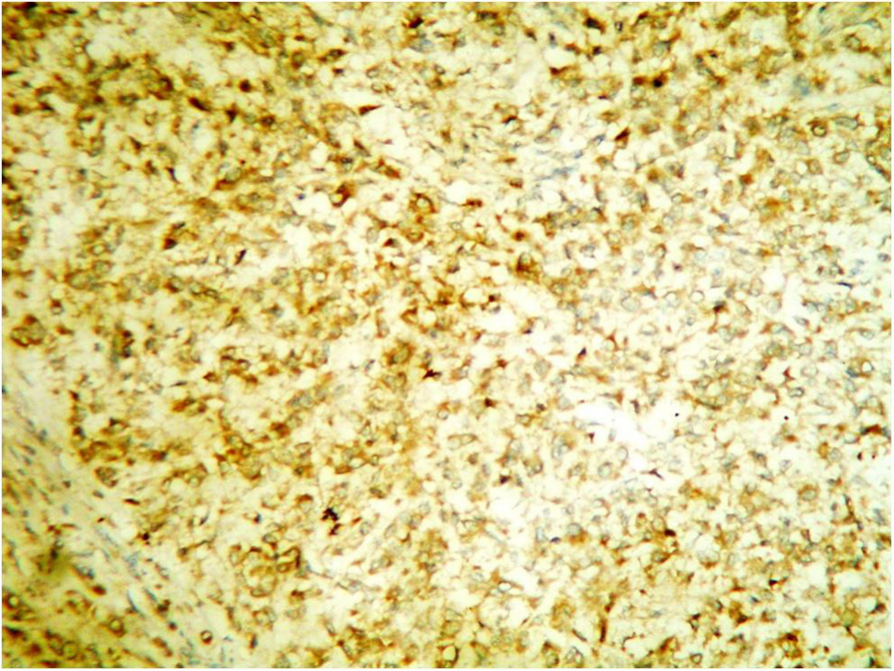
Tumor cells were positive for alpha-fetoprotein staining, ×20.

**Figure 6:**
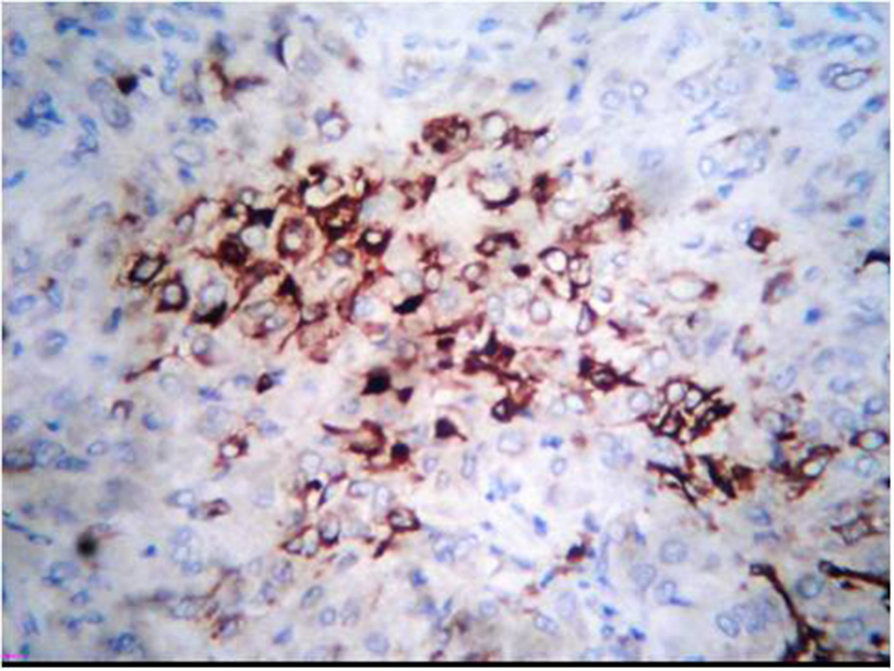
Tumor cells showed focal positivity for inhibin alpha, ×40.

Laboratory values of a complete blood count and thyroid function tests were within the normal limits. Serum concentrations of estradiol were in the range of 280 to 375 pmol/L (normal <73 for prepubertal child); progesterone, 5.3 nmol/L (normal 0 to 1.3 nmol/L); testosterone, 38.9 pg/mL (reference value 1 to 5.2 pg/mL); androstenedione, 4.1 ng/mL (normal, 0.3 to 3.1 ng/mL); and prolactin, 233 mIU/L (normal < 460 mIU/L). Basal levels of luteinizing hormone (LH) and follicle-stimulating hormone (FSH) were low (0.4 and 0.7 IU/L, respectively), with no change in response to stimulation by 100 mg of LH-releasing hormone intravenously. Regarding tumor markers, the total inhibin level was 1,069 U/L (normal <100 U/L for prepubertal child) and the alpha fetoprotein levelwas 987 μg/L (normal 1 to 200 μg/L) while B human chorionic gonadotropin, lactic dehydrogenase and alkaline phosphatase were negative. Her chromosomes were normal (46XX). Our patient underwent explorative laparotomy one week after her initial presentation to our department; a solid tumor localized to her right ovary was identified. A right salpingo-oophorectomy was performed. Gross examination showed that the tumor mass measured about 10 × 7 cm in diameter. The mass was capsulated and its outer surface was nodular with dilated congested blood vessels. The cut section was solid and with multiple cysts containing transparent thick material. The mass was firm in consistency with friable areas. Its color was yellow grayish with small dark areas of necrosis (Figure 2). The histopathological report was an MGCCST with a yolk sac tumor. The histological picture showed the presence of the yolk sac tumor and a Sertoli-Leydig cell tumor with intermediate differentiation (Figures 3, 4). An immunohistochemical study of the tumor cells showed diffuse positive staining for alpha-fetoprotein (Figure 5) and focal areas of positive staining for inhibin (Figure 6).

Our patient’s postoperative period course was unremarkable, although a short period of vaginal bleeding was noted a few days after surgery. Pubic hair was lost and her breasts became less prominent over the four weeks following surgery. One month after surgery, she was started on chemotherapy treatment with bleomycin, etoposide and cisplatin (BEP regimen) every three weeks for four cycles. Eight months after surgery and chemotherapy, she was tumor free on clinical examination and radiography; her estradiol and testosterone levels had decreased to 61 pmol/L and 3.1 pg/mL, respectively. Our plan is to follow her up every three months for two years then every six months for five years.

## Discussion

Children with precocious puberty may be stressed because of physical and hormonal changes they are too young to understand. Going through puberty early can also be difficult for a child emotionally and socially. Girls with precocious puberty may be confused or embarrassed about physical changes, such as getting their periods or having enlarged breasts well before any of their peers. Not only that, but precocious puberty could cause the child to become an object of adult sexual interest [9]. Idiopathic CPP carries no increased risk of mortality however, distinguishing between children with idiopathic CPP and rare patients with a central nervous system, adrenal or ovarian tumor is important because the latter group may be at risk for tumor-related complications [10].

Our index case presented with a typical picture suggestive of idiopathic CPP with maintenance of the harmony (consonance) of normal puberty, that is, breast and pubic hair development, vaginal bleeding, growth acceleration and normal magnetic resonance imaging of the brain. But, the presence of an ovarian mass that was confirmed by computed tomography and low levels of FSH and LH pointed to an ovarian tumor as the sole cause of her precocious puberty. Her advanced bone age and accelerated height were seen because of tumor-derived estradiol. Advanced bone age, especially if it has progressed beyond the height age, serves as a nonspecific biomarker of abnormal sex steroid production [11].

The endocrine effects of the tumor on our patient were probably dependent upon the stromal components and proportions of different elements. Tumors containing both Sertoli and Leydig cells have variable effects, depending partly upon the proportion of these tumor elements and the degree of histologic differentiation. Signs of androgen excess or overt virilization occur in about half of patients with Sertoli-Leydig cell tumors. As with the pure Leydig cell tumors, estrogenic manifestations also occur. Patients with Sertoli-Leydig cell tumors who have defeminizing or virilizing clinical signs invariably have elevated blood testosterone levels [12]. In addition to testosterone, plasma androstenedione and, less commonly, dehydroepiandrosterone, are occasionally elevated [13]. Focal immunohistochemical positivity for inhibin confirmed the presence of the sex cord-stromal element. Total inhibin is a sensitive immunohistochemical marker of ovarian sex cord-stromal tumors [14]. The high levels of estrogen account for the breast development observed in this child and the build-up of the endometrium [15]. We presume, based on the elevated serum level of progesterone, that our patient's tumor was intermittently releasing progesterone, causing her vaginal bleeding [16]. The development of pubic hair may be accounted for by the combination of elevated testosterone, androstenedione and estrogen levels that were most likely derived from the tumor [15].

An MGCCST with a yolk sac tumor of the ovary should be differentiated clinically and pathologically from a gonadoblastoma, described by Talerman in 1972 [17,18]. An MGCCST with a yolk sac tumor of the ovary is very rare and there have been no reported cases in Egypt. Most cases are found in infants and children younger than 10 years, but rare cases occur in adults [19]. By comparison, a gonadoblastoma is a well-recognized tumor that occurs almost exclusively in a patient with abnormal gonadal development and a karyotype containing Y-chromosome material [20]. The prognosis of MGCCST with a yolk sac tumor of the ovary is dependent on the response to therapy of the yolk sac tumor as well as other components [21].

## Conclusion

Although in most of girls with precocious puberty, the etiology is idiopathic, MGCCST with a yolk sac tumor of the ovary should be considered in the differential diagnosis for a prepubescent girl with an abdominal mass.

## Consent

Written informed consent was obtained from the patient’s parents for publication of this case report and any accompanying images. A copy of the written consent is available for review by the Editor-in-Chief of this journal.

## Abbreviations

CPP, central precocious puberty; FSH, follicle-stimulating hormone; LH, luteinizing hormone; MGCCST, mixed germ cell-sex cord-stromal tumor.

## Competing interests

The authors declare that they have no competing interests.

## Authors’ contributions

KA carried out the patient diagnosis, investigation, follow-up, management and writing of the manuscript. DS carried out the histopathological diagnosis and writing of the manuscript. HS carried out the patient diagnosis, follow-up, management and writing of the manuscript. HA carried out the patient diagnosis and follow-up. MA carried out the excision of the tumor. All authors read and approved the final manuscript.
